# Noble metal-comparable SERS enhancement from semiconducting metal oxides by making oxygen vacancies

**DOI:** 10.1038/ncomms8800

**Published:** 2015-07-17

**Authors:** Shan Cong, Yinyin Yuan, Zhigang Chen, Junyu Hou, Mei Yang, Yanli Su, Yongyi Zhang, Liang Li, Qingwen Li, Fengxia Geng, Zhigang Zhao

**Affiliations:** 1 Key Lab of Nanodevices and Applications, Suzhou Institute of Nano-Tech and Nano-Bionics, Chinese Academy of Sciences (CAS), Suzhou 215123, China; 2 Key Laboratory for Ultrafine Materials of Ministry of Education, School of Materials Science and Engineering, East China University of Science and Technology, Shanghai 200237, China; 3 College of Chemistry, Chemical Engineering and Materials Science, Soochow University, Suzhou 215123, China

## Abstract

Surface-enhanced Raman spectroscopy (SERS) represents a very powerful tool for the identification of molecular species, but unfortunately it has been essentially restricted to noble metal supports (Au, Ag and Cu). While the application of semiconductor materials as SERS substrate would enormously widen the range of uses for this technique, the detection sensitivity has been much inferior and the achievable SERS enhancement was rather limited, thereby greatly limiting the practical applications. Here we report the employment of non-stoichiometric tungsten oxide nanostructure, sea urchin-like W_18_O_49_ nanowire, as the substrate material, to magnify the substrate–analyte molecule interaction, leading to significant magnifications in Raman spectroscopic signature. The enrichment of surface oxygen vacancy could bring additional enhancements. The detection limit concentration was as low as 10^−7^ M and the maximum enhancement factor was 3.4 × 10^5^, in the rank of the highest sensitivity, to our best knowledge, among semiconducting materials, even comparable to noble metals without ‘hot spots'.

## Abstract (web summary)

Surface-enhanced Raman spectroscopy is widely used for rapid and sensitive molecular detection in chemistry and biology, but typically relies on noble metals. Here the authors report a non-stoichiometric semiconducting material with defect-rich surface that displays excellent detection limits and enhancement factors.

## Graphical abstract

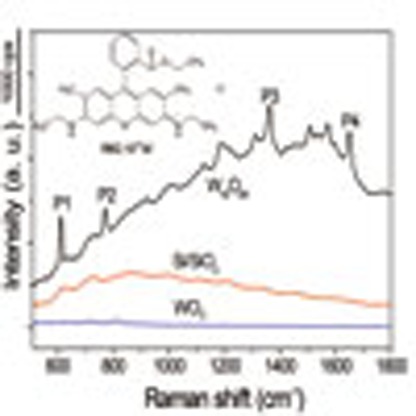

The surface-enhanced Raman scattering (SERS) effect is a surface-sensitive technique characterized by an increase in the Raman intensity by orders of magnitude for molecules adsorbed on particular surfaces such as rough metals with respect to that expected from the same number of non-adsorbed molecules in solution or the gas phase^1^,^2^. The enhanced sensitivity makes the technique serve as one of the most powerful analytical tools for the unequivocal identification of chemical and biological analytes and additionally in numerous other fields, such as electrochemistry, surface science, catalysis, chemical and biomolecular sensing and so on^1^,^3^,^4^. While the exact mechanism for the enhancement effect is still a matter of debate, two widely accepted theories have been adopted in most cases to explain the phenomenon^5^. The primary one is electromagnetic mechanism predicting that the electric field is magnified when excitation takes place within localized surface plasmon resonances of substrate materials, leading to enhancement factors (EFs) up to the order of 10^6^. The induced enhancement of local field by plasmonic coupling is called electromagnetic ‘hot spots'. The other is a chemical mechanism proposing the formation of charge-transfer complexes between the chemisorbed species and the substrate materials and obtaining enhancement when the excitation frequency is in resonance with the charge-transfer transition with EFs around 10–100. SERS experiments have been essentially dominated by adsorbates on rough metallic surfaces, especially noble and alkali metals such as Au, Ag and Cu, because their plasmon resonance frequencies locate within excitation wavelength ranges commonly used in Raman spectroscopy. While precise tuneable position of adjacent metallic nanoparticles is a key factor to bring about strong plasmonic coupling, fabricating perfect plasmonic nanostructures with high density ‘hot spots' to achieve high enhancements usually requires delicate procedures and high cost. In addition, noble metals typically show poor stability and biocompatibility, and therefore the search of other alternative materials for working as SERS substrate becomes an urgent task. A few semiconducting materials have been proven to show Raman enhancement, for example, InAs/GaAs quantum dots^6^, CuTe nanocrystals^7^, Cu_2_O nanospheres^8^ and TiO_2_ nanostructures^9^,^10^, in which charge transfer at the semiconductor–analyte interface plays a major role in Raman scattering enhancement. The TiO_2_ SERS substrate offers not only a biocompatibility, but also a greater chemical and mechanical stability against variation in pH or temperature of the environment^9^. The effective utilization of semiconductor SERS-active substrates would greatly expand the applications of SERS in many fields, such as direct monitoring of interfacial chemical reactions on individual nanoparticles^11^. However, one significant problem of metal-free SERS-active substrates is that the EFs were typically quite low, usually in the range of 10–10^2^, far from sufficient for applications in biological and biomedical analysis and diagnosis. It is consequently one formidable challenge but greatly desirable to find efficient semiconductor SERS substrate materials and obtain EFs comparable to noble metals.

The surface states of substrate material and its interaction with analyte molecule is a key to the SERS effect. Among the numerous methods in tuning the surface states of semiconducting oxide materials, adjusting oxygen deficiency represents one main strategy and may work as an efficient and simple way to achieve the goal of high sensitivity comparable to that of noble metals. Tungsten oxide materials, a traditional semiconductor, have attracted considerable attention because they possess distinctive physical and chemical properties, which endow them to be effective candidates in a wide range of applications, covering photocatalysts, gas sensors and electrochromic devices^12^,^13^,^14^. Importantly, rich phases of substoichiometric composition (WO_3−*x*_) can be obtained by the reduction and formation of various types of defect structure, such as WO_2.72_ (W_18_O_49_), WO_2.8_ (W_5_O_14_), WO_2.92_ (W_25_O_73_) and so on^15^,^16^,^17^,which makes the system work as an ideal platform for investigating the effect of vacancies or defects on SERS phenomenon. WO_2.72_ (W_18_O_49_) with the largest vacancy has been reported as the only oxide that can be isolated in a pure form, which contains tungsten ions of mixed valency^16^,^18^.

Herein, employing vacancy-containing W_18_O_49_ as the substrate material, we achieve greatly enhanced SERS effect for the first time on function-rich tungsten oxide material, and the enhancement is further improved by creating surface deficiencies, which gives SERS EF as high as 3.4 × 10^5^ and 100–10,000 times higher than the previously reported values for most other semiconductor SERS-active substrates, and even comparable to noble metals without ‘hot spots'.

## Results

### Sample characterizations

**Figure 1:**
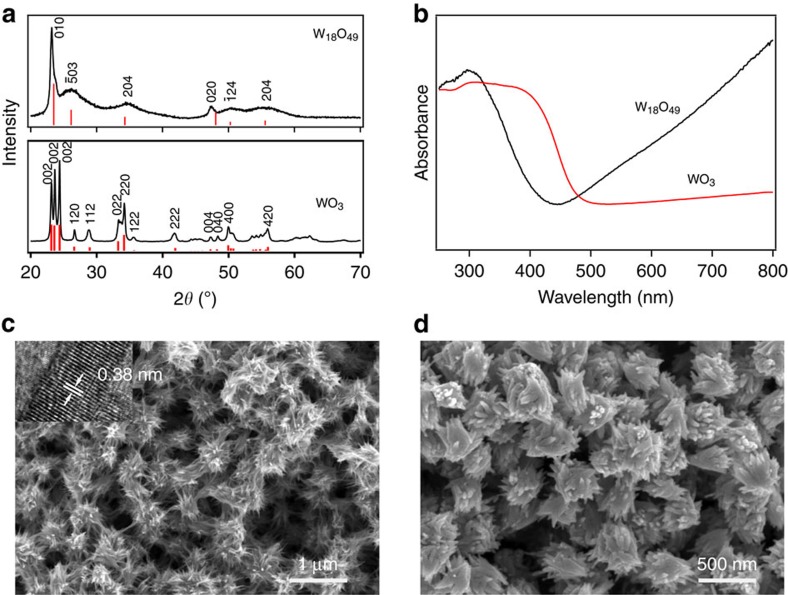
Characterization of W_18_O_49_ and annealed WO_3_ samples. (**a**) X-ray diffraction patterns of as-obtained W_18_O_49_ sample and annealed WO_3_ with all reflections perfectly indexed; (**b**) ultraviolet–visible profile comparison between W_18_O_49_ sample and annealed WO_3_, showing a blue shift and an obvious absorption tail beyond the edge that may arise from the presence of oxygen vacancies; (**c**,**d**) scanning electron microscopy images for W_18_O_49_ and WO_3_ samples, respectively. Inset in (**c**): high-resolution transmission electron microscopy image on one single nanowire illustrating clear lattice fringe of 0.38 nm, which suggested that the nanowire growth was along the [010] direction.

W_18_O_49_ was synthesized using a previously reported approach, WCl_6_ dissolved in ethanol followed by hydrothermal reaction at 180 °C for 24 h (ref. 13). Phase and purity of the sample was verified by X-ray diffraction characterizations as shown in Fig. 1a. The hydrothermally synthesized sample was crystallized in a monoclinic phase of W_18_O_49_ (*P2/m*, JCPDS no. 84-1516) with lattice constants refined to be *a*=18.31(2), *b*=3.839(8), *c*=14.00(1) Å and *β*=115.19(9)°. The relatively high intensities and narrowing exhibited by (010) and (020) peaks suggest that the crystals preferably grew along the *b* axis as a result of retarded growth along the close-packed (010) planes. For comparison study, tungsten trioxide, WO_3_, was acquired by annealing the W_18_O_49_ sample in air. The transformed WO_3_ was also of high purity with all reflections perfectly fitted in stoichiometric monoclinic (no. 14, *P2*_*1*_*/n*) tungsten trioxide with cell dimensions *a*=7.305(5), *b*=7.523(4), *c*=7.689(4) Å and *β*=90.90(9)°. To obtain more information about the intrinsic nature of the samples, ultraviolet–visible absorption spectra of the two samples were collected (Fig. 1b). Distinct from the absorption to 500 nm normally observed in tungsten oxide materials, W_18_O_49_ sample exhibited an obvious blue shift of the absorption edge. In addition, an absorption tail beyond the absorption edge in the visible and near-infrared region was observed, up to the measurement limit, 800 nm, which can be considered to be closely related with the free electrons and/or oxygen deficiency-induced small polarons^15^, providing clear evidence that the as-synthesized W_18_O_49_ sample contains a large number of oxygen vacancies. Morphology of the samples were characterized by scanning electron microscope observations, which suggested that the as-grown W_18_O_49_ sample comprised of thin nanowires with average length estimated to be about several micrometres while width in the range of 10–20 nm (Fig. 1c). The nanowires were entangled featuring a prickly, spherical and sea urchin-like morphology. High-resolution transmission electron microscopy on one single nanowire discerned clear lattice fringes belonging to (010) planes of monoclinic W_18_O_49_ with an interplanar spacing of ∼0.38 nm (inset in Fig. 1c), which undoubtedly indicates that the nanowires preferably grow along the [010] direction, in good agreement with X-ray diffraction observations. After annealing in air and oxidizing into WO_3_, the morphology was featureless, showing irregular particles of several micrometres (Fig. 1d).

**Figure 2:**
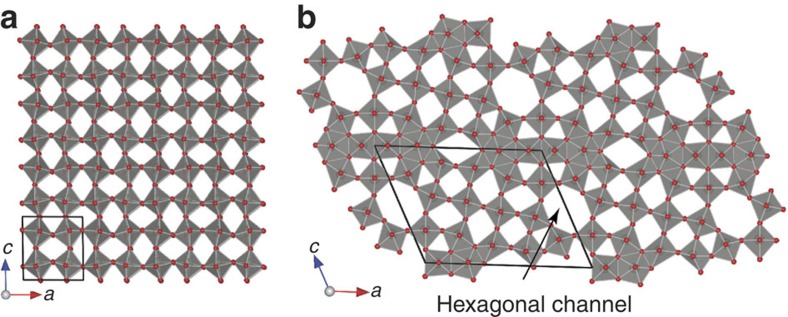
Structure illustration for WO_3_ and W_18_O_49_ in [010] projection. (**a**) The crystalline WO_3_ structure consists of stacking of infinite corner-sharing WO_6_ octahedra layers, while (**b**) the defects in W_18_O_49_ structure lead to the formation of hexagonal channels.

The defect structure of W_18_O_49_ is illustrated in Fig. 2, in comparison with the perfect structure of WO_3_, projected along the [010] direction. The crystal structure of WO_3_ consists of slabs of corner-sharing WO_6_ octahedra (ReO_3_-type), which have an infinite extension in two dimensions (*ac* plane) and a finite, characteristic width in the [010] direction. The slabs are mutually linked, forming channels between the octahedra. In contrast, the reduction of WO_3_ to W_18_O_49_ leads to structural changes of the ReO_3_-type structure and the formation of tungsten pentagonal columns causes the arising of additional hexagonal channels^19^. The presence of oxygen defects may enrich the surface states of semiconductor and enhance the interaction affinity between the adsorbent and adsorbate, providing promises for enhancements in Raman signals.

### Raman enhancement for W_18_O_49_ sample

**Figure 3:**
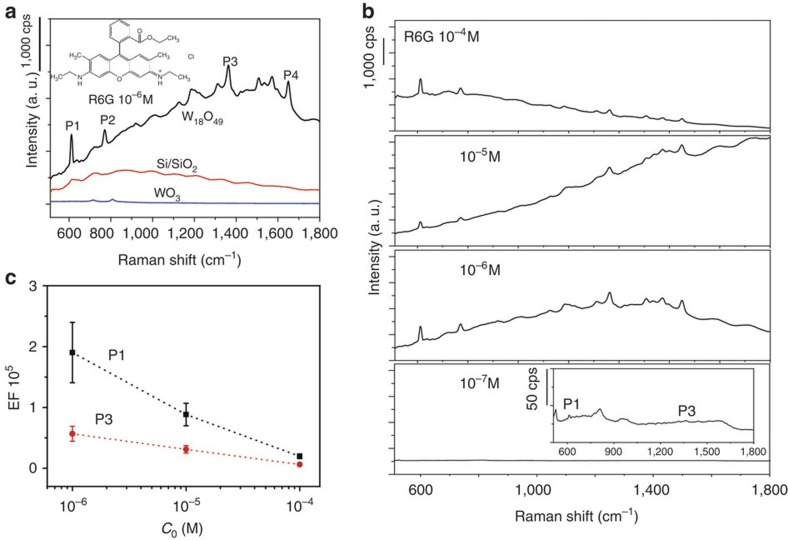
SERS properties. (**a**) Raman profile of R6G (10^−6^ M) on substrates deposited with W_18_O_49_ sample compared with that for WO_3_ and bare SiO_2_/Si substrate. Inset: molecule structure of R6G. (**b**) Raman spectra collected for W_18_O_49_ at four different concentrations, 10^−4^, 10^−5^, 10^−6^ and 10^−7^ M, suggesting the detection limit was as low as 10^−7^ M (Inset: with narrowed *y* scale for 10^−7^ M). (**c**) The statistical evolution of EF as a function of R6G concentration plotted in logarithmic scale, with the analysis carried out over 30 different regions per sample. The Raman enhancement typically increased with decreasing concentrations and selective enhancement occurred with different bands.

The Raman enhancement behaviour of the materials was examined using laser dye Rhodamine 6G (R6G) as a Raman probe, the molecular structure of which is provided in inset of Fig. 3a. R6G is a strongly fluorescent xanthene derivative that shows a molecular resonance effect when excited into its visible absorption band. Figure 3a shows the Raman spectra of R6G (10^−6^ M) with the excitation wavelength being 532.8 nm on substrates deposited with W_18_O_49_ nanowires, annealed WO_3_, along with bare SiO_2_/Si. While no clear signals related with R6G molecule were discerned on bare SiO_2_/Si substrate and WO_3_ except the fluorescence background, a substantial Raman enhancement was observed on W_18_O_49_, and four characteristic bands of R6G centred at 612, 773, 1,360 and 1,650 cm^−1^, named as P1, P2, P3 and P4, were clearly detected, which suggests that W_18_O_49_ could work as SERS-active substrate. Raman characterizations on bare W_18_O_49_ in the absence of probe molecules were also performed, in which only the signals related to O–W–O bending and W–O stretching modes were found (Supplementary Fig. 1), further confirming that the P1, P2, P3 and P4 bands originated from R6G molecules. P1 and P2 can be assigned to in-plane and out-of-plane bending motions of carbon and hydrogen atoms of the xanthenes skeleton, respectively; P3 and P4 correspond to aromatic C–C stretching vibration modes^20^. Raman spectra at four different R6G concentrations (the range was selected according to adsorption isotherms in Supplementary Fig. 2), decreasing from 10^−4^, 10^−5^ and 10^−6^ to 10^−7^ M, were collected, showing obvious intensity enhancement until an extreme dilute solution was used, 10^−7^ M, in which just detectable signal was obtained (Fig. 3b). Therefore, the detection limit for W_18_O_49_ material can be determined to be 10^−7^ M. Such a low detection limit enables a high sensitivity towards trace amounts of analyte molecules, and makes it possible for a systematic investigation of different vibration modes in the SERS profiles.

Two signature bands, P1 and P3, were selected for calculating EFs based on the magnification of Raman intensity compared with that on bare substrate (details in Supplementary Methods)^21^,^22^,^23^, the variation of which was depicted in Fig. 3c as a function of R6G analyte concentrations (*C*_R6G_). It is clear that the greater enhancement occurs for P1 at each concentration, which was found to be surprisingly high as 1.9 × 10^5^ with the R6G concentration of 10^−6^ M, about 100–10,000 times higher than that previously reported for most other semiconductor SERS-active substrates (Supplementary Table 1). Furthermore, the intensity variation was not uniform and instead strongly dependent on the band identity. The selective enhancement of the different vibration modes indicates that the SERS on W_18_O_49_ sample largely depends on the distinct binding and geometry of R6G molecules on the W_18_O_49_ surface, which is a character of the chemical enhancement mechanism. Specifically, the change of P1 was more obvious than that for P3. The relative lower intensity exhibited by P3, especially at low concentrations, indicates that the long axis plane of R6G molecule was not parallel to the sample surface or the aromatic rings were separated by chemical groups including C–H bond^24^. With dilution of the R6G solutions, the signal intensity of P3 gradually increases, suggesting the gradual decrease of tilt angles of R6G molecules on the substrate. The emergence of greatly enhanced SERS sensitivity for W_18_O_49_ compared with WO_3_ can probably be attributed to the presence of oxygen vacancies in W_18_O_49_ structure (*vide ante*, Fig. 2).

### Surface vacancy related Raman enhancement

**Figure 4:**
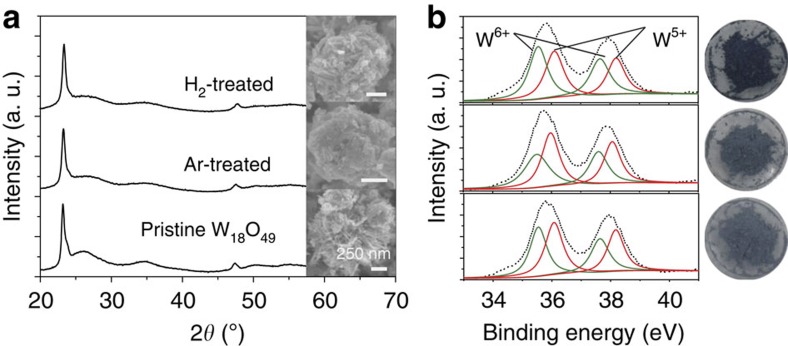
Characterization of W_18_O_49_ samples with modulated surface oxygen vacancies. (**a**) X-ray diffraction patterns of Ar- and H_2_-treated samples along with that for as-prepared W_18_O_49_ sample for comparison purpose, indicating that the crystalline phase remained during the annealing. Inset: the corresponding SEM images showing the morphology was also unchanged. (**b**) XPS spectra of W4f core levels for W_18_O_49_ samples after treatment in Ar and H_2_ atmosphere at 300 °C and pristine W_18_O_49_ sea urchin-like aggregates. Right: corresponding optical images of the three samples, showing colour change from pale blue for pristine W_18_O_49_ to cyan and deep blue for Ar- and H_2_-treated samples.

To confirm the role of oxygen vacancy played in SERS, additional surface vacancy was deliberately created by annealing the W_18_O_49_ sample in Ar/H_2_ atmosphere^25^. After the treatment at 300 °C for 1 h, the crystalline phase of W_18_O_49_ and the sea urchin-like morphology were still well kept (Fig. 4a). X-ray photoelectron spectroscopy was used to check the surface states, and Fig. 4b displays the spectra of W4f core levels for pristine W_18_O_49_ and the Ar/H_2_-treated samples. The W4f core-level spectra could be deconvoluted into three doublets (W^6+^, W^5+^ and W^4+^) for all samples. On the fitting analysis, it was found that the atomic percentage of W^6+^, W^5+^ and W^4+^ for pristine W_18_O_49_ was 54.1, 30.4 and 15.5%, roughly agreeing the documented values^26^. By contrast, the percentage for W^5+^ increased to 35.4 and 47.5%, W^4+^ to 21.4 and 13.9% for Ar and H_2_ thermal-treated samples, respectively. The increased percentage for reduced tungsten, W^4+^ and W^5+^, is likely to be related with the creation of positively charged oxygen vacancies with accompanying charge-compensating electrons^27^. With increasing oxygen vacancy, the intensity of the absorption tail in the visible and near-infrared region increased (Supplementary Fig. 3), consistent with the recorded results^28^. The sample colour changed from pale blue for pristine W_18_O_49_ to cyan and deep blue for Ar- and H_2_-treated samples, respectively, confirming the increment of oxygen deficiencies present in the examined samples.

**Figure 5:**
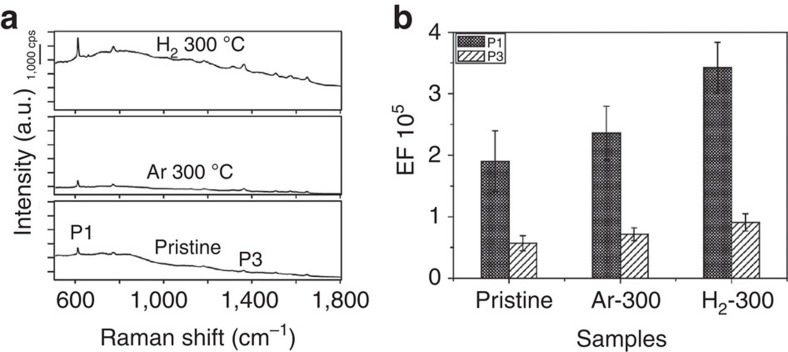
Improved SERS properties of W_18_O_49_ samples after Ar/H_2_ annealing treatment. (**a**) Raman signals of R6G molecule on pristine W_18_O_49_ and the samples after annealing treatment (in Ar/H_2_ at 300 °C for 1 h). The tested concentration of R6G was 1 × 10^−6^ M. (**b**) A comparison of Raman EF for the two respective vibration modes P1 and P3. Data reported in this histogram resulted from Raman spectra acquired over 30 different regions per sample and provide an indication of the EF for each Raman mode. The H_2_-treated sample shows the greatest enhancement at band P1, the EF for which was evaluated to be 3.4 × 10^5^.

The Raman spectra of R6G with concentration of 1 × 10^−6^ M for the two treated samples are given in Fig. 5a along with the spectrum of pristine W_18_O_49_ sample for comparison. Both treated samples clearly manifested the four characteristic peaks of R6G molecule. Neither a new peak nor an obvious shift of the characteristic peak was detected, indicating that no formation or vanish of chemical bonds between R6G and the substrates were induced on oxygen vacancy alternation. Notably, the Ar- and H_2_-treated samples were found to display further Raman intensity increase on some degree compared with pristine W_18_O_49_ sample, corroborating the fact that oxygen vacancy may help increase Raman detect sensitivity. The Raman intensity of the four feature bands for all the three samples were summarized in Fig. 5b, and it is obvious that the Raman enhancement was in the order of H_2_-treated W_18_O_49_>Ar-treated W_18_O_49_>pristine W_18_O_49_. The P1 band of H_2_-treated W_18_O_49_ displayed the greatest enhancement, and the corresponding EF was evaluated to be 3.4 × 10^5^. Such high EF has been rarely observed in semiconducting materials, even comparable to that for noble metals without hot spots^20^, which may come from the combined function of intrinsic and deliberately created oxygen deficiencies.

**Figure 6:**
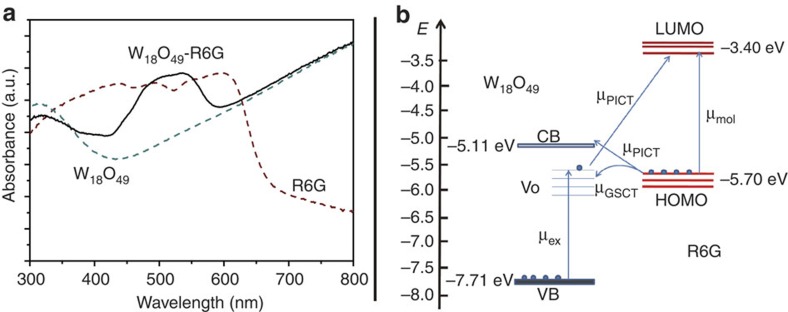
Charge transfer between R6G and W_18_O_49_. (**a**) Absorption spectra for R6G on W_18_O_49_ compared with neat W_18_O_49_ and R6G dye. (**b**) Energy-level diagram of R6G on oxygen-deficit W_18_O_49_ measured in a vacuum.

It seems the presence of oxygen vacancies in semiconducting oxide materials, either intrinsic ones or deliberately created on the surface, could bring significant Raman enhancement for R6G molecule. To examine the interaction between tungsten oxide materials with adsorbate molecule, ultraviolet–visible spectrum of R6G molecules deposited on W_18_O_49_ was collected in comparison with those for neat R6G and W_18_O_49_, which is presented in Fig. 6a. The spectrum for the hybrid features occurrence of a new band with an optical absorption onset at ∼580 nm. The band locates between the band–band transition absorption edge for W_18_O_49_, 420 nm, and the photoabsorption threshold for R6G dye, 670 nm, providing clear evidence for the efficient charge transfer between R6G and W_18_O_49_ material. This observation of high Raman enhancement on W_18_O_49_ may be based on the charge-transfer mechanism in an adsorbate–semiconductor system.

Moreover, oxygen vacancy may play an irreplaceable role in enriching the surface states of semiconductor to provide magnified affinity for the adsorbent–adsorbate interaction, which results in a further increase to the Raman signals for the probe molecules adsorbed on the semiconductor substrate surface through charge transfer involved in a CM mechanism. Providing with efficient charge transfer processes between the matching energy levels of adsorbed probe molecules and the semiconductor, both the polarizability tensor and the electron density distribution of the molecule would be modified, leading to the observation of non-totally symmetric SERS modes. As shown in Fig. 6b, R6G as a typical SERS probe molecule has the highest-occupied molecular orbital and lowest-unoccupied molecular orbital levels at −5.70 and −3.40 eV, respectively^29^. The valence band and conduction band of semiconductor W_18_O_49_ locate at −7.71 and −5.11 eV (ref. 30),respectively, with oxygen vacancy-associated electronic state (*V*_O_) well separated from the bottom of the CB, located at about 0.5–1.0 eV below the conduction band minimum^31^. It can be expected that contributions from several types of thermodynamically feasible charge transfer resonance may be related to the overall Raman enhancement in our W_18_O_49_–R6G system at an excitation of 532 nm, including molecule resonance (μ_mol_) of R6G, exciton resonance (μ_ex_) of W_18_O_49_ defect states, and the photon induced charge transfer resonance (μ_PICT_) together with the ground-state charge transfer resonance (μ_GSCT_) from matched energy level between W_18_O_49_ and R6G molecules. These resonances will lead to a magnification of Raman scattering cross-section.

In fact, defect state |*V*〉 stemming from oxygen vacancies in the lattice of semiconductor has a crucial contribution to the molecular polarizability tensor via the vibronic coupling with molecular ground state |*I*〉 and molecular excited states |*K*〉, besides the conduction band state |*S*〉 and valence band state |*S*′〉 of semiconductor (details are illustrated in Supplementary Methods). Under the incident laser frequency higher than the molecular resonance (*ω*_0_>*ω*_IK_), the polarizability tensor *α*_σρ_ can be expressed by a simple formula *α*_σρ_=*A*+*B*+*C*^32^, where *A* represents the contribution of the molecular resonance. *B* and *C* represent the contributions from the photoinduced charge transfer of the molecule-to-semiconductor and semiconductor-to-molecule, respectively, which are both related to the defect states in the semiconductor. It can be evidenced that additional possible resonant contribute to the total enhancement in defect-rich semiconductor–molecule system, compared with its non-defect counterpart. When these predicted multiplicatively coincide, large EFs are expected (Supplementary Fig. 4). Thus, the observed high SERS enhancement indicates that modulation of the surface vacancy of semiconductor substrate is a simple but effective means in design of a molecule-semiconductor system with high SERS enhancement.

## Discussion

In summary, metal-free SERS substrate with highly sensitive detection has been successfully fabricated employing non-stoichiometric tungsten oxide, W_18_O_49_ as an example, which achieved a limit detection as low as 10^−7^ M and EF up to 3.4 × 10^5^, an outstanding observation for semiconducting materials and even comparable to noble metal without ‘hot spots'. The oxygen vacancy played a critical role in amplifying the spectroscopic signatures of probe molecules, since tungsten trioxide, WO_3_, only gave extremely weak signals. Moreover, the artificial creation of oxygen deficiencies by annealing the material substrate in inert or reducing atmosphere (Ar/H_2_) brought about further Raman enhancements, providing unambiguous evidence that the presence of oxygen vacancy, either intrinsic or post-created ones, could help magnify the Raman signals. The extremely high SERS sensitivity on semiconducting materials can probably be attributed to the presence of oxygen deficiencies, either intrinsic or on the surface, and the resultant strengthened interaction with probe molecules via vibronic coupling. These observations provide important clues in the future strategy design of efficient semiconducting SERS substrate, and may bring important fundamental advance in material science and chemistry.

## Methods

### Synthesis of sea urchin-like W_18_O_49_ nanowires and WO_3_ nanoparticles on Si/SiO_2_

W_18_O_49_ nanowires in sea urchin-like morphology on Si/SiO_2_ substrate were prepared according to a recently published hydrothermal approach^13^. In brief, WCl_6_ (0.099 g) was dissolved in 30 ml absolute ethanol, which was transferred to a Teflon-lined stainless steel autoclave holding several horizontally oriented Si/SiO_2_ substrates. The autoclave was then sealed and heated at 180 °C for 12 h. After finishing the reaction, the substrates were rinsed thoroughly with absolute ethanol and dried naturally at room temperature before use. The W_18_O_49_ nanowires on substrate were transformed to the corresponding trioxide form, WO_3_, by annealing in air. The temperature was set at 500 °C and the sample was kept at the temperature for 1 h.

### Modulating surface vacancy states

Modulation of the surface oxygen vacancy states was achieved through annealing the as-prepared W_18_O_49_ nanowires by heating in Ar/H_2_ atmosphere at 300 °C for 1 h. The velocity of Ar/H_2_ was fixed at 400 and 200 ml min^−1^, respectively.

### Raman measurement

To study the Raman enhancement effect by the tungsten oxide materials, R6G dissolved in deionized water was employed to be the probe molecule because R6G has a large Raman scattering cross-section at the laser excitation wavelength applied for the SERS experiments, 532.8 nm (ref. 33). The tested concentration ranged from 10^−4^ to 10^−7^ M. Stock solution of 10^−3^ M was initially made, and solutions of other concentrations were obtained by successive dilution by factors of 10^1^ or 10^2^. After dropping an aliquot of the respective solutions on the substrate and drying for at least 5 h, Raman spectra were subsequently collected on a high-resolution confocal Raman spectrometer (LabRAM HR-800) using the same instrumental settings for ready comparisons. The excitation wavelength was 532.8 nm and a × 50 L objective was used to focus the laser beam. The spectra were acquired for 30 s with three accumulations and the laser power was maintained at 0.3 mW with an average spot size of 1 μm in diameter in all acquisitions. For each sample, Raman spectra from different areas were collected, and the signal intensity was averaged for final analysis, from which relative s.d. values for EFs were estimated (Supplementary Methods and Supplementary Figs 5–8).

## Additional information

**How to cite this article:** Cong, S. *et al*. Noble metal-comparable SERS enhancement from semiconducting metal oxides by making oxygen vacancies. *Nat. Commun.* 6:7800 doi: 10.1038/ncomms8800 (2015).

## Supplementary Material

Supplementary Information Supplementary Figures 1-8, Supplementary Table 1, Supplementary Methods and Supplementary References
