# Supplementary material for: Noble metal-comparable SERS enhancement from semiconducting metal oxides by making oxygen vacancies
Source: Nat Commun. 2015 Jul 17;6:7800. doi: 10.1038/ncomms8800 (PMC4518302; doi:10.1038/ncomms8800)
Supplement: Supplementary Information — Supplementary Figures 1-8, Supplementary Table 1, Supplementary Methods and Supplementary References [file ncomms8800-s1.pdf]

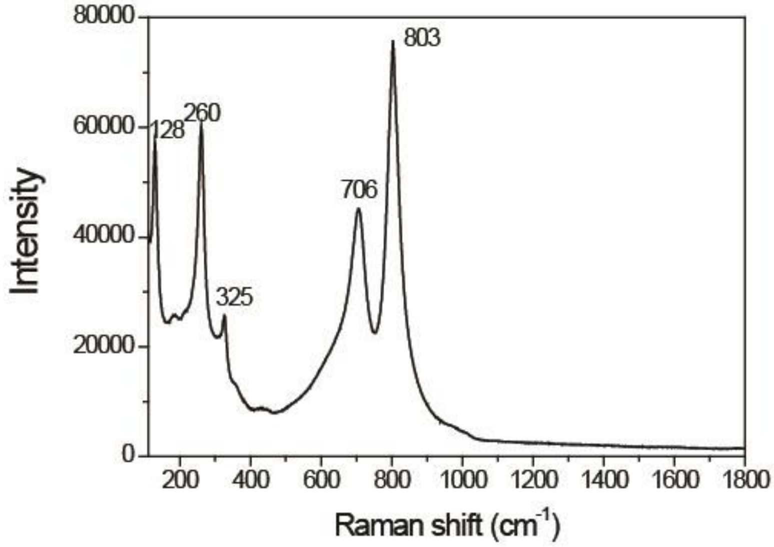

Supplementary Figure 1 | Raman profile for bare $W_{18}O_{49}$ sample.

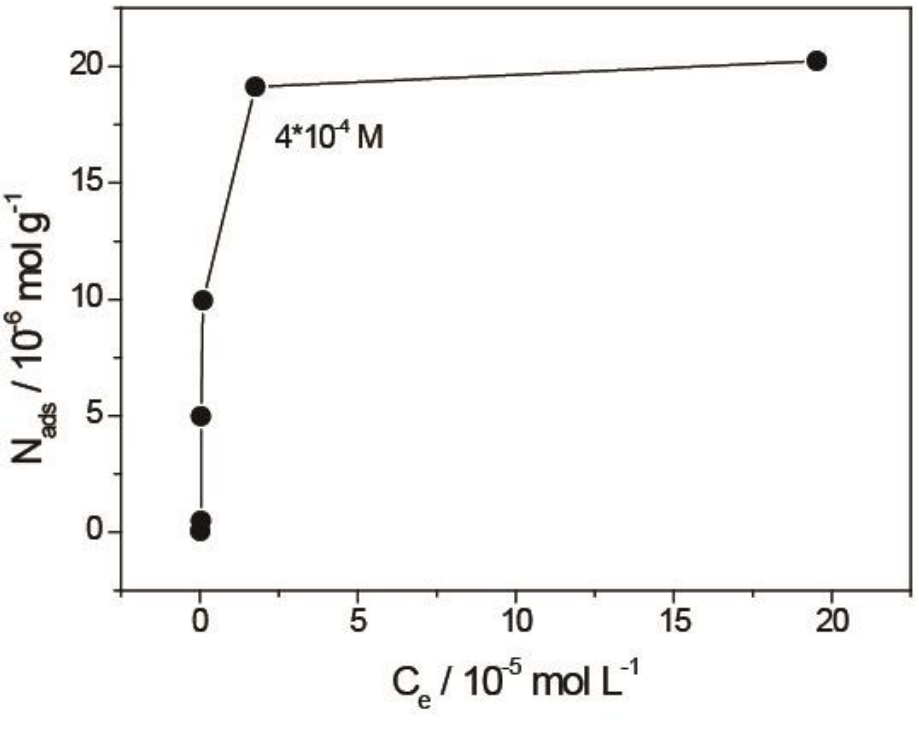

Supplementary Figure 2 | R6G adsorption isotherms. Adsorbed amount versus equilibrium concentration onto as-prepared $W_{18}O_{49}$ sample. For an initial concentration of R6G lower than $4*10^{-4}$ M, the maximum amount of R6G adsorbed onto $W_{18}O_{49}$ substrate (effective substance concentration of about 2 mg/ml) is not reached.

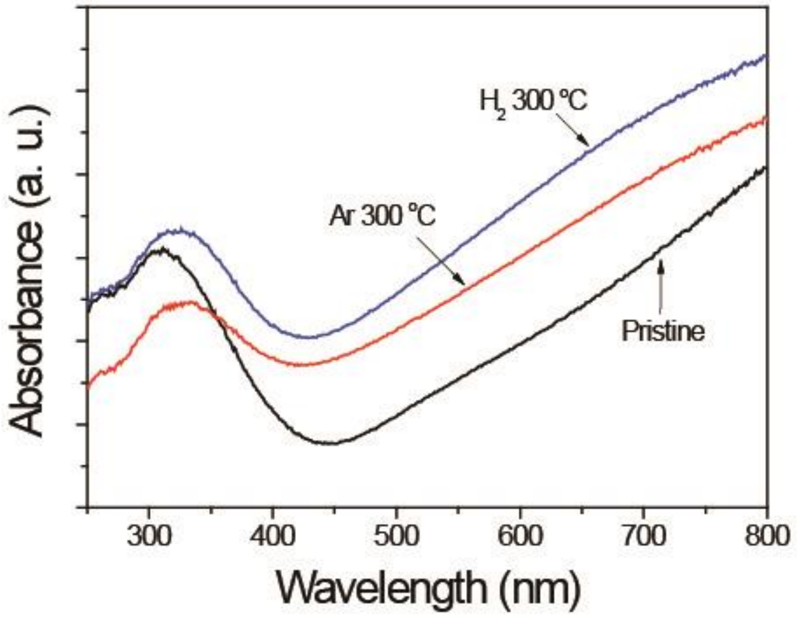

Supplementary Figure 3 | UV-vis absorption spectra. Ar- and H_2-treated samples along with that for as-prepared $W_{18}O_{49}$ sample.

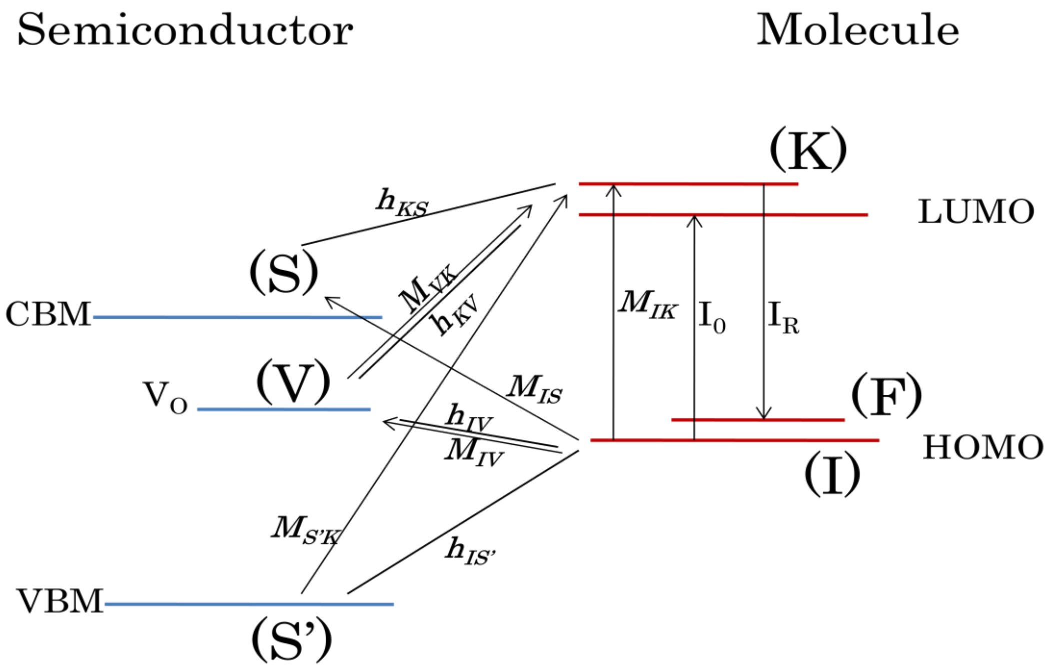

Supplementary Figure 4 | The scheme of charge transfer in a semiconductor-molecule system. The photo-induced charge transfer of semiconductor-to-molecule and molecule-to-semiconductor under the situation of $\omega_0 > \omega_{IF}$ for a defect-rich semiconductor.

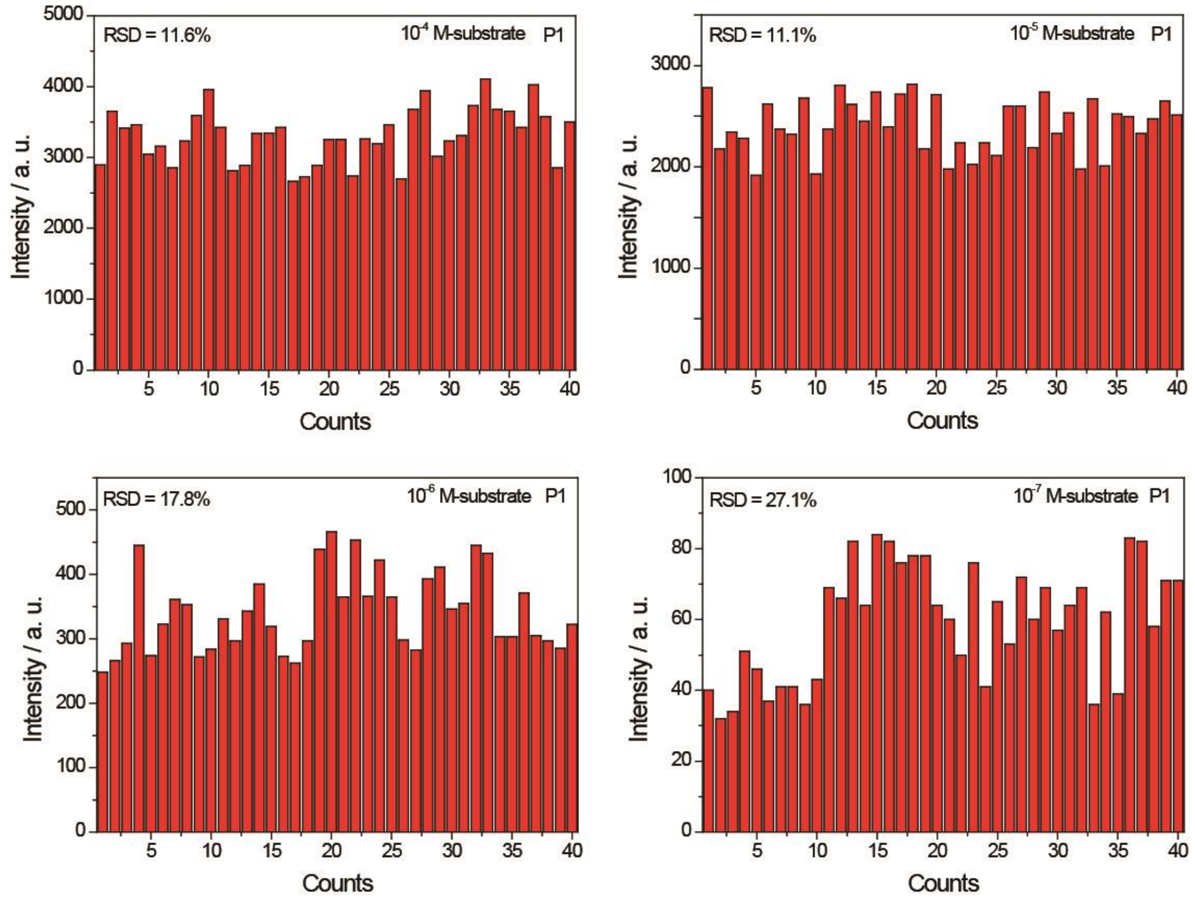

Supplementary Figure 5 | The intensities of the P1 (612 cm^{-1}) Raman vibration mode of R6G on varied substrates. Four different concentrations, 10^{-4}, 10^{-5}, 10^{-6}, and 10^{-7} M, were examined with data acquired by recording 40 spectra from eight different substrates (five stochastic spots per substrate). The RSD of the P1 (612 cm^{-1}) Raman vibration mode of R6G at four different concentrations, 10^{-4}, 10^{-5}, 10^{-6}, and 10^{-7} M, are calculated to be 11.6%, 11.1%, 17.8% and 27.1%, respectively.

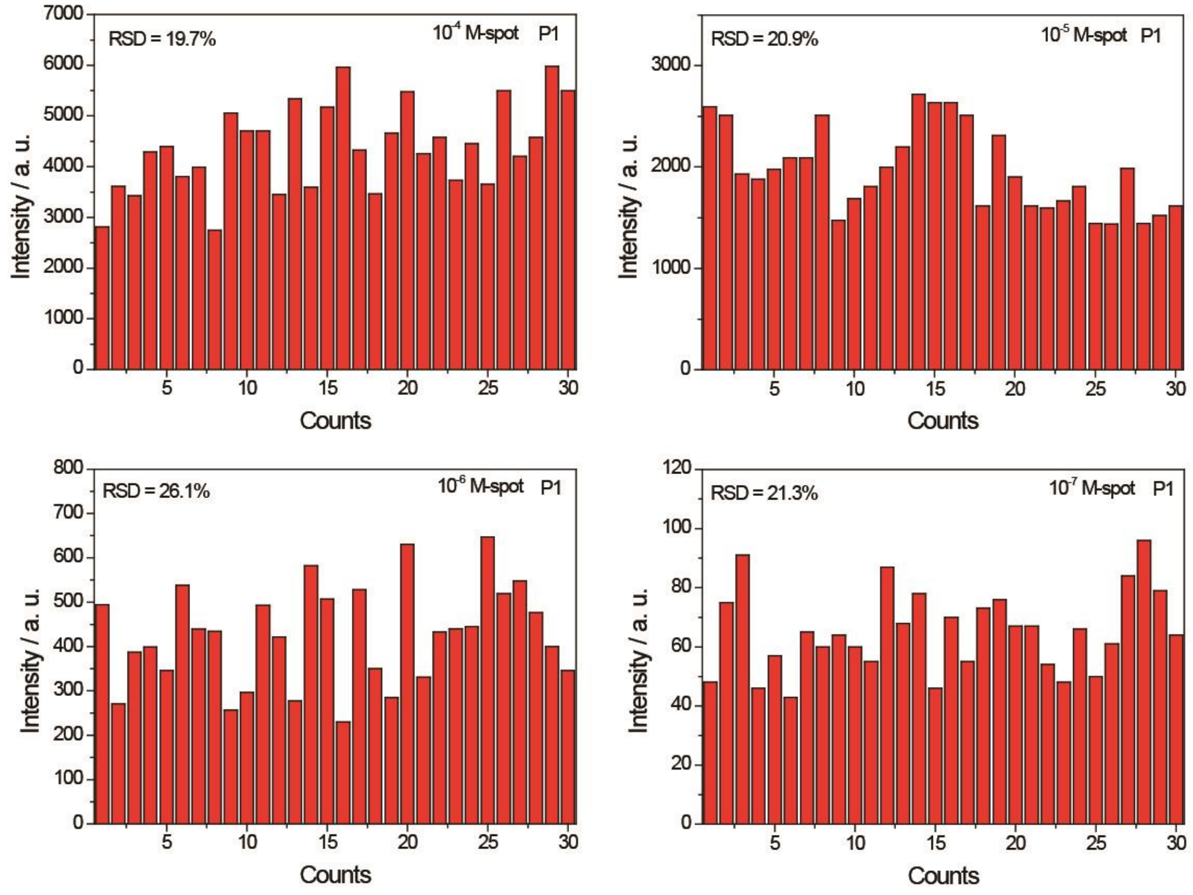

Supplementary Figure 6 | The intensities of the P1 (612 cm^{-1}) Raman vibration mode of R6G on varied spots. Four different concentrations, 10^{-4}, 10^{-5}, 10^{-6}, and 10^{-7} M, were examined with data acquired by recording spectra at 30 stochastic spots at different locations across from one substrate. The RSD of the P1 (612 cm^{-1}) Raman vibration mode of R6G at four different concentrations, 10^{-4}, 10^{-5}, 10^{-6}, and 10^{-7} M, are calculated to be 19.7%, 20.9%, 26.1% and 21.3%, respectively.

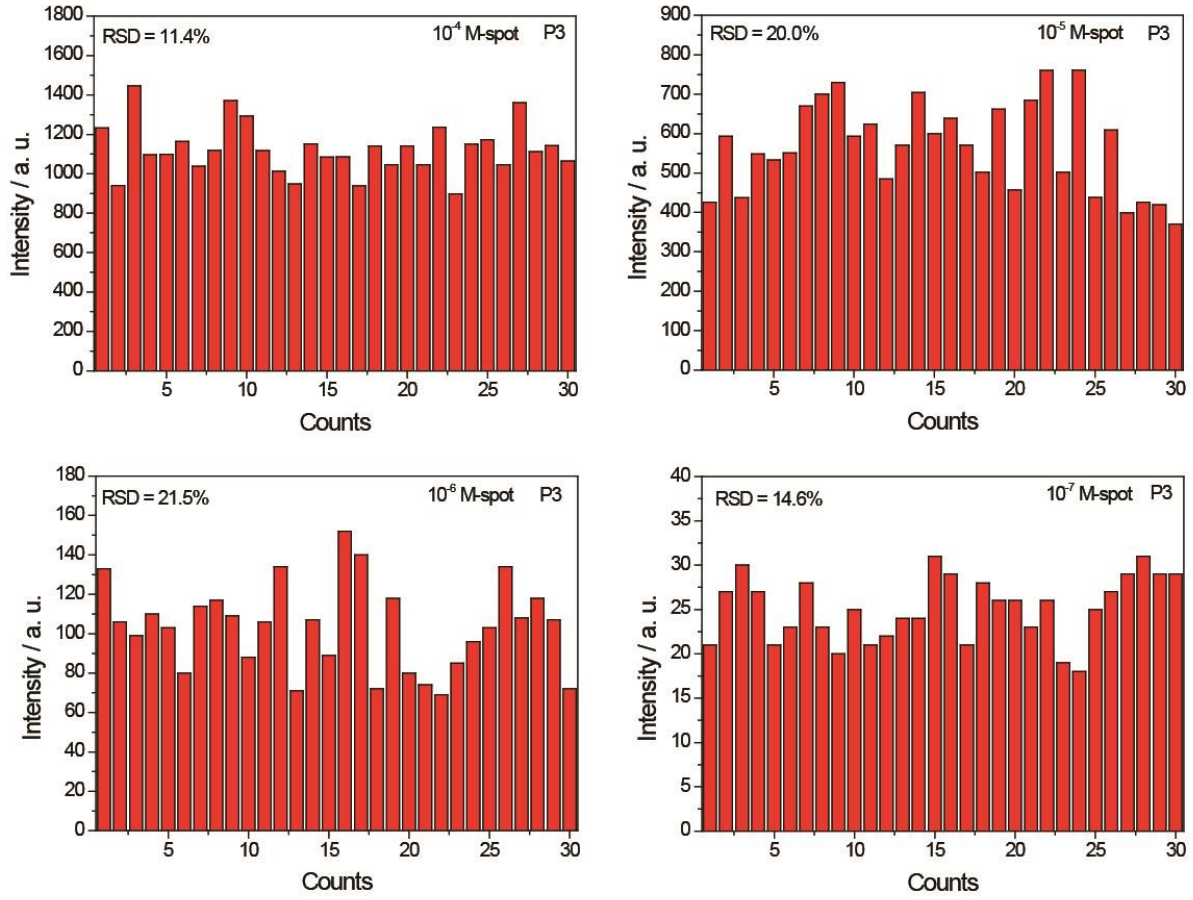

Supplementary Figure 7 | The intensities of the P3 (1360 cm^{-1}) Raman vibration mode of R6G on varied spots. Four different concentrations, 10^{-4}, 10^{-5}, 10^{-6}, and 10^{-7} M, were examined with data acquired by recording spectra at 30 stochastic spots at different locations across from one substrate. The RSD of the P3 (1360 cm^{-1}) Raman vibration mode of R6G at four different concentrations, 10^{-4}, 10^{-5}, 10^{-6}, and 10^{-7} M, are calculated to be 11.4%, 20.0%, 21.5% and 14.6%, respectively.

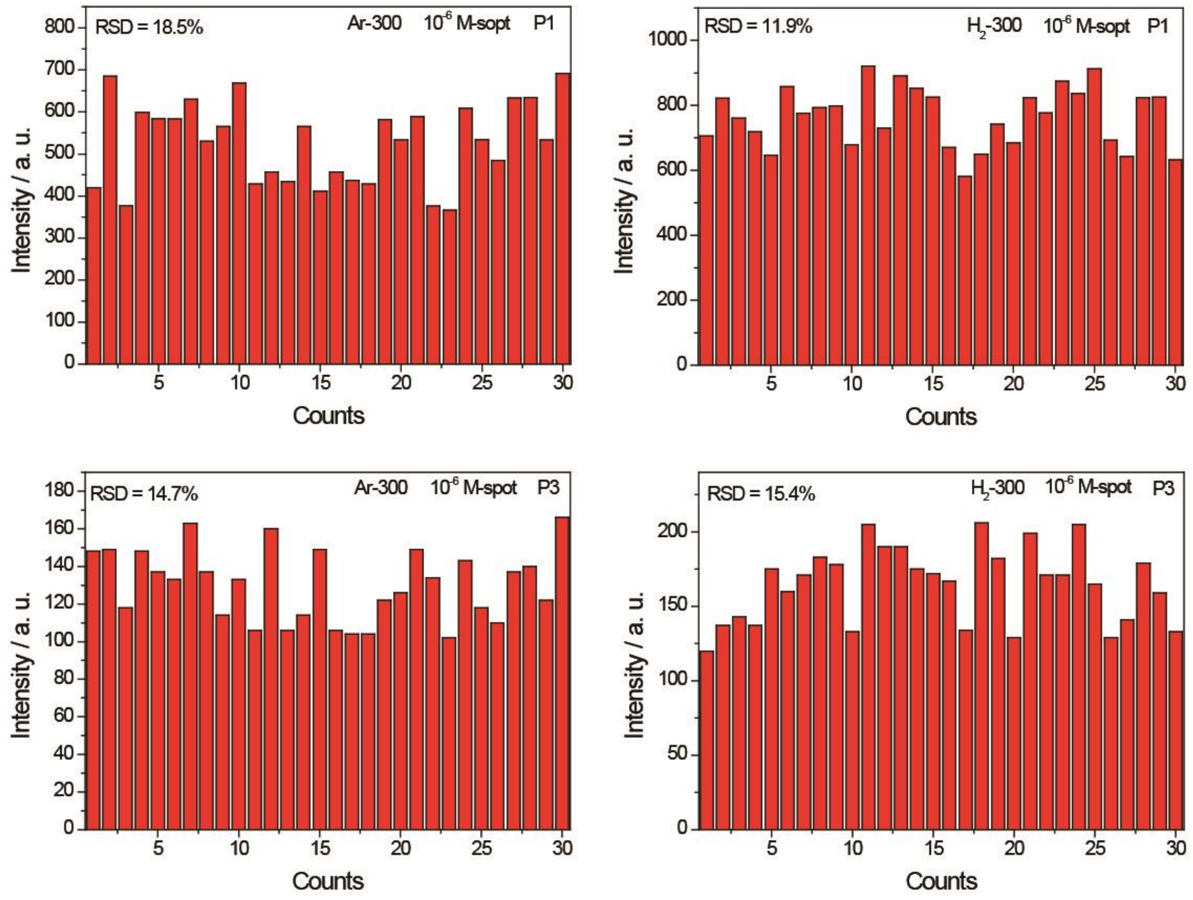

Supplementary Figure 8 | The intensities of Raman signals of P1 (612 cm^{-1}) and P3 (1360 cm^{-1}) vibration modes of R6G molecule. $W_{18}O_{49}$ substrates were pre-treated under Ar or H_2 atmosphere at 300 ^oC for 1h. The tested concentration of R6G was 10^{-6} M. Data were acquired by recording spectra at 30 stochastic spots at different locations across from one substrate. The RSD of the P1 (612 cm^{-1}) and P3 (1360 cm^{-1}) vibration modes of R6G molecule (10^{-6} M) are calculated to be 18.5%, 14.7% on Ar-treated $W_{18}O_{49}$ samples, and 11.9%, 15.4% for H_2-treated $W_{18}O_{49}$ samples.

Supplementary Table 1 | Performance of SERS active materials from literatures. Both noble metals without 'hot spot' and semiconducting materials are included.[1-9]

Reference	Material	Analyte	EF	Detection Limit (M)	Excited wavelength (nm)
P. Hildebrandt et. al.	Colloidal Ag	R6G	10^6	10^{-9}	514
K. Kim et al.	ZnO nanorod arrays	4-ABT	22	10^{-3}	514.5, 632.8
Y. Wang et al.	ZnO nanocrystals	4-Mpy	10^3	10^{-3}	514.5
D. Qi et al.	TiO_2 photonic microarray	MB	$2*10^4$	$6*10^{-6}$	532
Y. Wang et al.	CuO	4-Mpy	10^2	10^{-3}	514.5
L. Jiang et al.	Cu_2O nanospheres	4-MBA	10^5	10^{-3}	488
S. Hayashi et al.	GaP	Cu Pc	700	---	514.5
L. G. Quagliano	InAs/ GaAs quantum dots	pyridine	10^3	---	514.5
X. Wang et al.	H-Si nanowire	R6G	8~28	10^{-6}	532
**this work**	**$W_{18}O_{49}$**	**R6G**	**$3.4\pm0.41\times10^5$**	**10^{-7}**	**532.8**

Supplementary Methods

Characterizations. The surface morphologies of $W_{18}O_{49}$ analogues were measured using a FEI Quanta 400 FEG field emission scanning electron microscope (SEM). TEM images were obtained employing FEI Tecnai G2 F20 S-Twin at 200 kV. XRD patterns of the prepared samples were recorded on a Bruker AXS D8 Advance X-ray diffractometer with a Cu Kα radiation target (40V, 40A). UV-Vis experiments were made in transmission geometry on a UV-Vis spectrophotometer (V660, JASCO) over a wavelength range of 200-800 nm.

Calculation of the enhancement factor. The EF was calculated according to the formula:

$$EF = (I_{\mathrm{SERS}}/N_{\mathrm{SERS}})/(I_{\mathrm{bulk}}/N_{\mathrm{bulk}}) \quad (1)$$

$$N_{\mathrm{SERS}} = CVN_{\mathrm{A}}A_{\mathrm{Raman}}/A_{\mathrm{Sub}} \quad (2)$$

$$N_{\mathrm{bulk}} = M\rho hA_{\mathrm{Raman}}N_{\mathrm{A}} \quad (3)$$

I_{SERS} and I_{bulk} are the intensities of the selected Raman peak in the SERS and non-SERS spectra, and N_{SERS} and N_{bulk} are the average number of molecules in scattering area for SERS and non-SERS measurement. The data for R6G (0.05 M) on bare Si/SO_2 substrate were used as non-SERS-active reference. Specifically, the intensity was obtained by taking average from measurements of 30 spots, and the number of analyte molecules was estimated by Supplementary equation 2 on the assumption that the analyte molecules were distributed uniformly on the substrates. C is the molar concentration of the analyte solution, V is the volume of the droplet, N_{A} is Avogadro constant. A_{Raman} is the laser spot area (1 μm in diameter) of Raman scanning. Twenty microliters of the droplet on the substrate was spread into a circle of about 3 mm in diameter after solvent evaporation, from which the effective area of the substrate, A_{Sub}, can be obtained. The confocal depth (h) of the laser beam is 21 μm,[10] and on the basis of molecular weight (M) and density (ρ) of bulk R6G (1.15 g cm^{-3}), N_{bulk} is calculated by Supplementary equation 3.

The uniformity and reproducibility for statistical analysis. The uniformity was acquired by recording spectra at 30 stochastic spots at different locations across one substrate. The reproducibility between different substrates was acquired by recording 40 spectra from eight different substrates (five stochastic spots per substrate). The peak at 612 cm^{-1} (P1) and 1360 cm^{-1} (P3) of R6G Raman spectrum were chosen for quantification because of their relative insensitivity and the representative for different type of vibrations.

The calculation of the contribution of photo-induced charge transfer (PICT) to the molecule polarization tensor in the defect-rich semiconductor-molecule system. Based on the Herzberg-Teller theory regarding the vibronic coupling of the zero-order Born-Oppenhermer states, the contribution of photo-induced charge transfer (PICT) to the molecule polarization tensor in the semiconductor-molecule system can be estimated. The calculation in this communication is derived from the treatment in a semiconductor-molecule system reported by Lombardi and Wang,[9,11] respectively, except for the difference that we include the vibronic coupling of the defect states $|V\rangle$ in with the molecular exited state $|K\rangle$ and the molecular ground state $|I\rangle$ in the semiconductor-molecule system.

The intensity of a Raman transition may be obtained from the molecular polarizability tensor by the expression:

$$I_{\mathrm{Raman}} = \left[\frac{8\pi(\omega_0 \pm \omega_{\mathrm{IF}})^4 I_0}{9c^4}\right] \sum \alpha_{\rho\sigma}^2 \tag{4}$$

Where I_0 is the incident laser intensity at frequency ω_0, and ω_{IF} is a molecular transition frequency between states I and F (presumably two different vibronic levels of the ground electronic state I_e).

The general expression for the polarizability tensor in the molecule-semiconductor system may be shown as:

$$\alpha_{\sigma\rho} = \sum_{K\neq I,F}\left(\frac{\langle I|\mu_\sigma|K\rangle\langle K|\mu_\rho|F\rangle}{E_K - E_I - \hbar\omega_0} + \frac{\langle I|\mu_\rho|K\rangle\langle K|\mu_\sigma|F\rangle}{E_K - E_F + \hbar\omega_0}\right) \quad (5)$$

Where *K* represents all the other states of the molecule, μ is the dipole moment operator, and σ, ρ are the scattered and incident polarization directions in space (**X**, **Y**, **Z**). Following Lombardi and Wang, using the zero-order Born-Oppenheimer approximation, all the vibronic states (*I*, *K*, *F*) as products of the electronic and vibrational wave functions can be written as follows:

$$|I\rangle = I_e\rangle|i\rangle,\ |K\rangle = K_e\rangle|k\rangle,\ |F\rangle = I_e\rangle|f\rangle \quad (6)$$

Where the subscript *e* indicates a purely electronic state, and lower case letters represent vibrational functions. Based on the Herzberg-Teller theory that even small vibrations may cause mixing of zero-order Born-Oppenheimer states, vibronic functions in a defect-rich semiconductor-molecule system can be written as:

$$|K_e\rangle = |K_e, 0\rangle + \sum_S \lambda_{KS} Q\,|S_e, 0\rangle + \sum_V \lambda_{KV} Q\,|V_e, 0\rangle \quad (7)$$

$$|I_e\rangle = |I_e, 0\rangle + \sum_{S'} \lambda_{IS'}\,Q|S'_e, 0\rangle + \sum_V \lambda_{IV} Q\,|V_e, 0\rangle \quad (8)$$

$$\lambda_{KS} = \frac{h_{KS}}{(E_K^0 - E_S^0)} = h_{KS}/\omega_{KS} \qquad \lambda_{KV} = \frac{h_{KV}}{(E_K^0 - E_V^0)} = h_{KV}/\omega_{KV}$$

$$\lambda_{IS'} = \frac{\lambda_{IS'}}{\left(E_I^0 - E_{S'}^0\right)} = h_{IS'}/\omega_{IS'} \qquad \lambda_{IV} = \frac{\lambda_{IV}}{(E_I^0 - E_V^0)} = h_{IV}/\omega_{IV} \quad (9)$$

$$h_{KS} = \left\langle K_e, 0\left|\frac{\partial H_{eN}}{\partial Q}\right|S_e, 0\right\rangle \qquad h_{KV} = \left\langle K_e, 0\left|\frac{\partial H_{eN}}{\partial Q}\right|V_e, 0\right\rangle$$

$$h_{IS'} = \left\langle I_e, 0\left|\frac{\partial H_{eN}}{\partial Q}\right|S'_e, 0\right\rangle \qquad h_{IV} = \left\langle I_e, 0\left|\frac{\partial H_{eN}}{\partial Q}\right|V_e, 0\right\rangle \quad (10)$$

Where *zero* refers to zero-order Born-Oppenheimer states, H_{eN} is the electron-nuclear attraction term in the Hamiltonian, evaluated at the equilibrium nuclear positions (0). $|S_e\rangle$ and $|S_e'\rangle$ indicates the electronic states lying in conductor band and valence band of semiconductor, respectively. h_{KS} is the coupling matrix element representing the degree to which a particular vibration Q can mix states S_e with state K_e. Similarly, $h_{IS'}$ is the coupling matrix element representing the degree to which a particular vibration Q can mix states S_e' with state I_e.

It should be mention that in a defect-rich semiconductor, surface or lattice defects such as oxygen vacancies can introduce new electronic states, located in the forbidden band, which should be also included in the vibronic functions. The defect states may serve as an electron sink, with a dual ability to trap and eject excitons from/to matched energy levels. With a similar approach, we use $|V_e\rangle$ to denote the electronic state related to the defects lying in the forbidden band of semiconductor. h_{KV} is the coupling matrix element representing the degree to which a particular vibration Q can mix states V_e with state K_e, and h_{IV} is the coupling matrix element representing the degree to which a particular vibration Q can mix state V_e with state I_e.

For the purely electronic transition moment between states, we write:

$$M_{IK} = \langle I_e|\mu|K_e\rangle, \quad M_{S'K} = \langle S'_e|\mu|K_e\rangle, \quad M_{IS} = \langle I_e|\mu|S_e\rangle,$$
$$M_{IV} = \langle I_e|\mu|V_e\rangle, \quad M_{VK} = \langle V_e|\mu|K_e\rangle. \tag{11}$$

Substituting into Supplementary equation 5 (assuming that $\omega_0 > \omega_{IF}$), the expression of polarizability tensor $\alpha_{\sigma\rho}$ in the defect-rich semiconductor-molecule system can be derived, which still involves the sum of three terms：

$$\alpha_{\sigma\rho} = A + B + C \tag{12}$$

$$A = \sum_{K_e \neq I_e} \sum_k \left[\frac{M_{IK}^{\sigma} M_{KI}^{\rho}}{\hbar(\omega_{KI} - \omega_0)} + \frac{M_{IK}^{\rho} M_{KI}^{\sigma}}{\hbar(\omega_{KI} + \omega_0)} \right] \langle i|k\rangle \langle k|f\rangle$$

（13）

$$\begin{aligned} B = & \sum_{K_e \neq I_e} \sum_k \sum_{S_e \neq K_e} \left\{ \left[\frac{h_{KS} M_{IK}^{\sigma} M_{SI}^{\rho}}{\hbar(\omega_{KI} - \omega_0)} + \frac{h_{KS} M_{IK}^{\rho} M_{SI}^{\sigma}}{\hbar(\omega_{KI} + \omega_0)} \right] \frac{\langle i|k\rangle \langle k|Q|f\rangle}{\hbar\omega_{KS}} \right. \\ & \left. + \left[\frac{h_{KS} M_{IS}^{\sigma} M_{KI}^{\rho}}{\hbar(\omega_{KI} - \omega_0)} + \frac{h_{KS} M_{IS}^{\rho} M_{KI}^{\sigma}}{\hbar(\omega_{KI} + \omega_0)} \right] \frac{\langle i|Q|k\rangle \langle k|f\rangle}{\hbar\omega_{KS}} \right\} \\ & + \sum_{K_e \neq I_e} \sum_k \sum_{V_e \neq K_e} \left\{ \left[\frac{h_{KV} M_{IK}^{\sigma} M_{VI}^{\rho}}{\hbar(\omega_{KI} - \omega_0)} + \frac{h_{KV} M_{IK}^{\rho} M_{VI}^{\sigma}}{\hbar(\omega_{KI} + \omega_0)} \right] \frac{\langle i|k\rangle \langle k|Q|f\rangle}{\hbar\omega_{KV}} \right. \\ & \left. + \left[\frac{h_{KV} M_{IV}^{\sigma} M_{KI}^{\rho}}{\hbar(\omega_{KI} - \omega_0)} + \frac{h_{KV} M_{IV}^{\rho} M_{KI}^{\sigma}}{\hbar(\omega_{KI} + \omega_0)} \right] \frac{\langle i|Q|k\rangle \langle k|f\rangle}{\hbar\omega_{KV}} \right\} \end{aligned}$$

（14）

$$\begin{aligned} C = & \sum_{K_e \neq I_e} \sum_k \sum_{S'_e \neq I_e} \left\{ \left[\frac{h_{IS'} M_{IK}^{\sigma} M_{KS'}^{\rho}}{\hbar(\omega_{KI} - \omega_0)} + \frac{h_{IS'} M_{IK}^{\rho} M_{KS'}^{\sigma}}{\hbar(\omega_{KI} + \omega_0)} \right] \frac{\langle i|k\rangle \langle k|Q|f\rangle}{\hbar\omega_{IS'}} \right. \\ & \left. + \left[\frac{h_{IS'} M_{S'K}^{\sigma} M_{KI}^{\rho}}{\hbar(\omega_{KI} - \omega_0)} + \frac{h_{IS'} M_{S'K}^{\rho} M_{KI}^{\sigma}}{\hbar(\omega_{KI} + \omega_0)} \right] \frac{\langle i|Q|k\rangle \langle k|f\rangle}{\hbar\omega_{IS'}} \right\} \\ & + \sum_{K_e \neq I_e} \sum_k \sum_{V_e \neq I_e} \left\{ \left[\frac{h_{IV} M_{IK}^{\sigma} M_{KV}^{\rho}}{\hbar(\omega_{KI} - \omega_0)} + \frac{h_{IV} M_{IK}^{\rho} M_{KV}^{\sigma}}{\hbar(\omega_{KI} + \omega_0)} \right] \frac{\langle i|k\rangle \langle k|Q|f\rangle}{\hbar\omega_{IV}} \right. \\ & \left. + \left[\frac{h_{IV} M_{VK}^{\sigma} M_{KI}^{\rho}}{\hbar(\omega_{KI} - \omega_0)} + \frac{h_{IV} M_{VK}^{\rho} M_{KI}^{\sigma}}{\hbar(\omega_{KI} + \omega_0)} \right] \frac{\langle i|Q|k\rangle \langle k|f\rangle}{\hbar\omega_{IV}} \right\} \end{aligned}$$

（15）

A represents the contribution of the molecular resonance to the polarizbility tensor via M_{IK}, which is a term insusceptible to the defect states in the semiconductor, as described by Lombardi and Wang.

B represents the contribution of photo-induced charge transfer of molecule-to-semiconductor to the polarizability tensor via M_{IS} and M_{IV}. The transition borrow intensity from the allowed transition M_{IK} by means of vibronic coupling between the excited molecular state *K* and semiconductor conductor band state *S* through the matrix element h_{KS}, similarly, the transition borrow intensity from the allowed transition M_{IK} by means of vibronic coupling between the excited molecular state *K* and semiconductor defect state *V* through the matrix element h_{KV}.

C represents the contribution of photo-induced charge transfer of semiconductor-to-molecule to the polarizability tensor via $M_{S'K}$ and M_{VK}. The transition borrow intensity from the allowed transition M_{IK} by means of vibronic coupling between the molecular ground state *I* and semiconductor valence band state *S'* through the matrix element $h_{IS'}$, similarly, the transition borrow intensity from the allowed transition M_{IK} by means of vibronic coupling between the molecular ground state I and semiconductor defect state *V* through the matrix element h_{IV}.

This is illustrated in the scheme of Supplementary Figure 4.

Supplementary References

1. Hildebrandt, P. & Stockburger, M. Surface-enhanced resonance Raman spectroscopy of Rhodamine 6G adsorbed on colloidal silver. *J. Phys. Chem.***88**, 5935-5944 (1984).

2. Kim, K.; Kim, K. L.; Shin, K. S. Raman spectral characteristics of 4-aminobenzenethiol adsorbed on ZnO nanorod arrays. *Phys. Chem. Chem. Phys.***15**, 9288-9294 (2013).

3. Wang, Y.; Ruan, W.; Zhang, J.; Yang, B.; Xu, W.; Zhao, B; Lombardi, J. R. Direct observation of surface-enhanced Ramanscattering in ZnO nanocrystals. *J. Raman Spectrosc.***40**, 1072-1077 (2009).

4. Qi, D.; Lu, L.; Wang, L.; Zhang, J. Improved SERS sensitivity on plasmon-free TiO_2 photonic microarray by enhancing light-matter coupling. *J. Am. Chem. Soc.***136**, 9886-9889 (2014).

5. Wang, Y.; Hu, H.; Jing, S.; Wang, Y.; Sun, Z.; Zhao, B.; C. Zhao,; Lombardi, J. R. Enhanced Raman scattering

as a probe for 4-mercaptopyridine surface-modified copper oxide nanocrystals. *Anal. Sci.* **23**, 787-791 (2007).

6. Jiang, L.; You, T.; Yin, P.; Shang, Y.; Zhang, D.; Guo, L.; Yang, S. Surface-enhanced Raman scattering spectra of adsorbates on Cu_2O nanospheres: charge-transfer and electromagnetic enhancement. *Nanoscale* **5**, 2784-2789 (2013).

7. Hayashi, S.; Koh, R.; Ichiyama,Y.; Yamamoto, K. Evidence for surface-enhanced Raman scattering on nonmetallic surfaces: Copper phthalocyanine molecules on GaP small particles. *Phys. Rev. Lett.***14**, 1085-1088 (1988).

8. Quagliano, L. G. Observation of molecules adsorbed on III-V semiconductor quantum dots by surface-enhanced Raman scattering. *J. Am. Chem. Soc.***126**, 7393-7398 (2004).

9. Wang, X.; Shi, W.; She, G.; Mu, L. Using Si and Ge nanostructures as substrates for surface-enhanced Raman scattering based on photoinduced charge transfer mechanism. *J. Am. Chem. Soc.***133**, 16518-16523 (2011).

10. Cai, W. B.; Ren, B.; Li, X. Q.; She, C. X.; Liu, F. M.; Cai, X. W.; Tian, Z.Q. Investigation of surface-enhanced Raman scattering from platinum electrodes using a confocal Raman microscope: dependence of surface roughening pretreatment. *Surf. Sci.* **406**, 9-22 (1998).

11. Lombardi, J. R.; Birke, R. L. Theory of surface-enhanced Raman scattering in semiconductors. *J. Phys. Chem.* **118**, 11120-11130 (2014).
